# Parent Understanding and Satisfaction with NICU Discharge for Infants with Medical Complexity: A Scoping Review of Discharge Instruction Tools

**DOI:** 10.21203/rs.3.rs-9930572/v1

**Published:** 2026-06-05

**Authors:** Lamia Alam, Daniel J. France

**Affiliations:** Vanderbilt University Medical Center; Vanderbilt University Medical Center

## Abstract

**Background:**

Infants with medical complexity discharged from the neonatal intensive care unit (NICU) often require technology-supported care and frequent follow-up. In this setting, discharge safety depends on parent caregivers’ understanding of instructions, confidence performing specialized tasks, and the reliability of post-discharge coordination. Structured discharge instruction approaches and digital tools are increasingly used to standardize education and reinforce learning.

**Objective:**

To map and synthesize evidence on parent caregiver–perceived understanding and satisfaction with NICU discharge instructions when structured or digital discharge instruction tools are used, with attention to evidence comparing infants with medical complexity with infants without medical complexity at discharge.

**Methods:**

A scoping review was conducted across PubMed, Embase, CINAHL, Scopus, and the Cochrane Library. English-language studies addressing NICU discharge instruction processes/tools and parent-reported understanding and/or satisfaction were included. After duplicate removal, 281 records were screened; 63 full texts were assessed, yielding 35 sources (32 peer-reviewed papers and 3 clinical trial registry records). Findings were synthesized thematically.

**Results:**

Evidence consistently framed NICU discharge as a longitudinal transition rather than a single event. Six themes emerged: (1) Caregiver readiness and skill-building; (2) Psychosocial adaptation; (3) Care coordination and continuity across systems; (4) Home care management; (5) Post-discharge surveillance and follow-up engagement; (6) Late preterm safety standards. Interventions and tools (e.g., structured education plans, written materials, family-centered models, telehealth-enabled discharge planning) were generally associated with improved caregiver preparedness and perceived readiness, but benefits varied by family needs and context.

**Conclusions:**

Structured and digital discharge tools may improve parent understanding and satisfaction, but they work best as part of a coordinated, needs-based transition. Across studies, readiness extended beyond clinical stability to include caregiver confidence and judgment, reliable handoffs, and plans that fit family capacity. Breakdowns clustered at outpatient transitions when guidance and follow-up were unclear. Priorities include stronger cross-setting coordination, post-discharge safety nets, and future research focusing on standardized measures and implementation-focused designs for NICU discharge planning for medically complex infants.

## 1. Introduction

Children with medical complexity (CMC) make up a small portion of the pediatric population but account for a disproportionate share of health care use and costs.^1–3^ Advances in neonatal and pediatric care have improved survival, leading to more infants who require intensive, technology-supported care from birth and continue to have complex needs after discharge. Many of these infants are cared for in the NICU and leave the hospital still dependent on durable medical equipment (e.g., feeding tubes, home oxygen, tracheostomy and suction support, and monitoring devices) and frequent follow-up.^4–6^

For medically complex NICU graduates, discharge is a high-stakes transition from continuous clinical surveillance to parent caregiver-managed care at home. Safety depends not only on medical stability, but also on parent caregiver readiness and self-efficacy to perform specialized tasks, recognize deterioration, and coordinate care across multiple outpatient services. Yet discharge teaching is often compressed into the final days, delivered across many clinicians, and occurring during a stressful period for families—conditions that can leave gaps in understanding and confidence despite “complete” instructions.^7^

Structured discharge instruction approaches (e.g., staged education plans, checklists, teach-back, competency verification) and digital tools (e.g., multimedia materials and telehealth-supported handoffs) are increasingly used to standardize education and reinforce learning. This scoping review synthesizes evidence on parent caregivers perceived understanding and satisfaction with NICU discharge when structured or digital discharge instruction tools are used, with attention to differences for medically complex infants compared with infants without medical complexity at the time of discharge. The objective of this review is to synthesize evidence on parental understanding and satisfaction with NICU discharge instructions, focusing on studies of medically complex infants and structured or digital discharge instruction tools.

## 2. Methods

### 2.1 Design

We reported this scoping review in accordance with the Preferred Reporting Items for Systematic Reviews and Meta-Analyses (PRISMA) extension for scoping reviews (PRISMA-ScR).^8^ We used the scoping review approach to map and summarize evidence across different study designs and intervention types.

### 2.2 PICOT question

Using a PICOT framework, we examined: **P**: Parents of medically complex infants in the NICU; **I**: Structured or digital discharge instruction tools; **C**: Parents of infants without medical complexity; **O**: Parent-perceived understanding and satisfaction with the discharge process; **T**: At the time of discharge.

### 2.3 Search database and strategy

Searches were conducted in PubMed, Embase, CINAHL, Scopus, and the Cochrane Library through November 2025. Search strategies combined controlled vocabulary (e.g., MeSH/Emtree/CINAHL Headings) and keyword terms across five concept blocks: (1) parents/caregivers, (2) NICU/newborn/prematurity, (3) hospital discharge and discharge education/instructions, (4) parent experience outcomes (e.g., satisfaction, perceptions, understanding/comprehension), and (5) infant medical complexity (e.g., chronic disease, complex chronic conditions, technology dependence). Where applicable, searches also included terms for infants without medical complexity (e.g., healthy/normal newborn) to capture comparative evidence. Searches were limited to English-language records. Complete database-specific search strings are provided in Appendix A.

### 2.4 Eligibility Criteria and Study selection

Eligible sources included primary research studies and relevant clinical trial registry records that addressed NICU discharge education, discharge instruction tools, or discharge planning processes for infants with medical complexity (e.g., technology dependence, extreme prematurity), with outcomes related to parent understanding, perceptions, or satisfaction at or near discharge. Studies focused exclusively on outcomes beyond the discharge period without linkage to discharge preparation were excluded. Additional exclusions included reviews, case reports without a discharge component, and studies not addressing discharge processes or discharge-related education/coordination.

**Figure 1:**
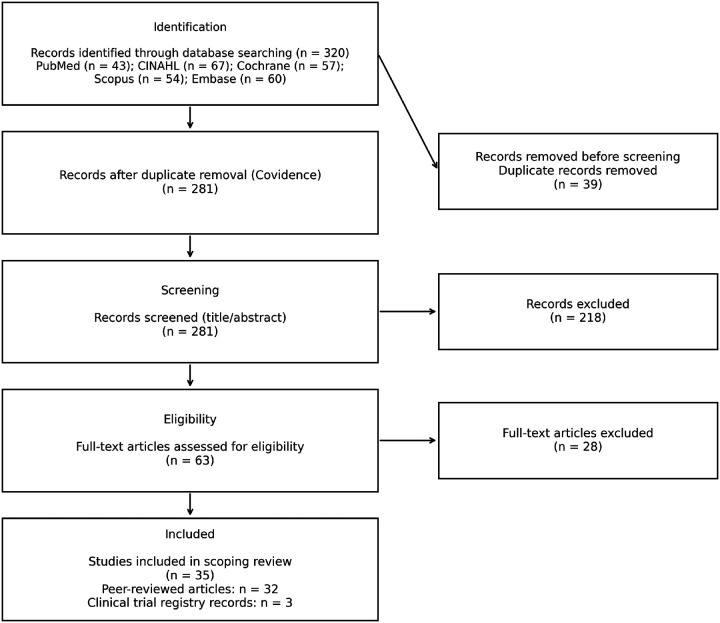
Flowchart of study search and screening.

Records were imported into Covidence for deduplication and screening. After duplicate removal, 281 records remained for title and abstract screening. One reviewer screened titles and abstracts using predefined exclusion criteria and excluded 218 records. Two reviewers independently assessed 63 full-text articles for eligibility and excluded 28 studies that did not meet inclusion criteria. Discrepancies were resolved through discussion and consensus. The final sample included 35 sources: 32 peer-reviewed papers and 3 clinical trial registry records (see Fig. 1). Registry records were treated as evidence-in-progress unless results were publicly available.

### 2.5 Data charting and synthesis

Key data elements were charted (study design, setting, study population or medical complexity, discharge tool or intervention, outcomes, and timing). Findings were synthesized using thematic analysis to identify cross-cutting patterns related to parent understanding and satisfaction.

## 3. Results

Across 32 papers, evidence included qualitative studies of caregiver experience, mixed-methods discharge transition evaluations, intervention studies, clinical reviews or guidelines, and implementation or protocol papers. The evidence consistently showed that NICU discharge is not a single event. Rather, it is a complex transition shaped by caregiver readiness and skills, their psychosocial adaptations during the transition, continuity of care, and the demands of medical complexity. Complete list of papers with the relevant themes is provided in Appendix B.

### Theme 1: Caregiver readiness and skill-building

A large portion of the literature emphasized that caregiver readiness is built through hands-on practice, progressive responsibility, and clear expectations. Parents defined readiness as doing care independently and gaining confidence.^9–11^ Similar readiness needs were reported in high-acuity contexts such as congenital heart surgery, where parents described anxiety about feeding, symptoms, and emergency decisions, even when formal teaching was delivered.^12^ Structured educational resources such as discharge booklets were positioned as tools to reduce information gaps and standardize teaching.^13^ Programs designed to strengthen readiness showed improvements in parent caregivers’ preparedness and stress outcomes,^14^ and system-level implementation work supported feasibility and workflow integration when family-centered discharge models were adopted.^15^ Interventions are increasingly being formalized for rigorous testing by defining multi-component family-centered care support and measurable outcomes, but promotion and implementation of the family-integrated care model for the discharge of medically complex infants from the NICU remains limited^16^. Qualitative study with families (i.e. semi-structured interview) also supported the value of sustained transition supports during early post-discharge life.^17^

### Theme 2: Psychosocial adaptation

Infants with medical complexity leaving the NICU undergo a vulnerable transition from continuous, team-based monitoring to complex home care directed primarily by parent caregivers. Many studies showed discharge is emotionally demanding, with families carrying fear, uncertainty, and identity disruption well beyond leaving the hospital. Formal support can help, but it is not universally beneficial, and may even increase distress for some families if mismatched to needs.^18^ Parents of medically complex infants described heavy psychological load, chronic uncertainty, and strain on family functioning.^19,20^ Similar emotional patterns appeared in congenital heart disease (CHD) hospital journeys, where communication and trust affected emotional burden for both parents and clinicians.^21^ Trauma-linked experiences were also described in families transitioning home after critical events like birth asphyxia, highlighting persistent fear and need for long-term emotional support.^22^ Technology dependence intensified the caregiving experience, including vigilance, isolation, and chronic stress,^23^ and social networks strongly shaped coping when infants were discharged with technology dependence.^24^ Grounded theory work reinforced that transition home often involves rebuilding identity and routines while managing uncertainty, emphasizing the importance of establishing good rapport between families and clinicians.^25^

### Theme 3: Care coordination and continuity across systems

Care continuity was repeatedly described as a vulnerable point for infants with medical complexity. Primary Care Providers (PCPs) reported inconsistent guidance and gaps in discharge communication for home oxygen and NG feed management, often relying on specialists without standardized handoffs.^26^ Telehealth supported discharge planning via video conferencing helped reduce access barriers and improved multidisciplinary alignment for complex discharges.^27^ Reviews emphasized that medically complex infants require structured care plans and proactive coordination in primary care to reduce risk and fragmentation.^28^ Community health visiting teams were described as critical bridge-supports, reinforcing teaching, assessing home safety, and helping families navigate care systems in the UK.^29^ Condition-specific qualitative work in severe bronchopulmonary dysplasia (BPD) showed that family social needs, health literacy, and system-level limitations directly impact follow-up quality and access, making coordination both essential and difficult.^30^ Finally, culturally aligned support frameworks highlighted that communication and discharge teaching must account for beliefs, meaning-making, and family coping patterns.^31^

### Theme 4: Home care management

Several papers focused on the complexity of managing chronic conditions and technology at home, including oxygen therapy, tube feeding, and preventive planning. BPD management was described as variable across settings and requiring clearer outpatient strategy and discharge planning.^32^ Home oxygen prescribing patterns showed inconsistencies that support the need for standardization and clearer weaning pathways.^33^ Discharge for technology-dependent preterm infants was framed as a high-risk transition requiring early equipment coordination, caregiver training, and structured follow-up planning.^34^ Tube feeding at home carried heavy routine and anxiety burdens, but parents gradually developed more expertise in care tasks, highlighting the value of accessible troubleshooting support.^35^ Discharge planning also included respiratory syncytial virus (RSV) prevention, stressing consistent prophylaxis decisions and clear parent education for high-risk premature infants.^36^ For infants with hydrocephalus, parents described long-term uncertainty and intensive navigation needs across evolving care episodes.^37^

### Theme 5: Post-discharge surveillance and follow-up engagement

Two papers specifically emphasized that safe transition requires sustained monitoring and reliable follow-up access. Parents of infants with severe BPD identified practical barriers to attending follow-up care, including transportation, schedule overload, and unclear perceived value, suggesting that follow-up engagement requires logistical and motivational supports.^38^ For CHD infants, structured home monitoring tools were developed to help families identify early deterioration and take measures appropriately, strengthening safety during the vulnerable post-discharge window.^39^

### Theme 6: Late preterm discharge safety standards

One guideline paper focused on discharge safety at the population level. Late preterm infants were framed as physiologically vulnerable after discharge, requiring standardized discharge criteria and early follow-up planning to reduce avoidable morbidity and readmissions.^40^

### Evidence from registered clinical trials (WHO ICTRP)

Three trials aligned with structured and/or digital discharge supports were identified from WHO ICTRP records:

1. **IRCT201604084613N19** evaluated an empowerment education program plus written/manual and multimedia materials, with outcomes including maternal satisfaction, self-efficacy, and discharge preparation measured near discharge.^41^
2. **IRCT2015062922978N1** evaluated a randomized discharge-planning program with multiple education sessions plus weekly post-discharge calls, assessing maternal quality of life and perceived social support at discharge and post-discharge.^42^
3. **ISRCTN27425533** evaluated a randomized web-based video communication intervention prior to neonatal back-transport, measuring parent-rated information quality and psychological preparation at discharge.^43^

No outcomes were posted in the registry records reviewed.

## 4. Discussion

This synthesis shows that NICU discharge planning is best understood as a longitudinal transition process rather than a single end-of-stay event. Across studies, parents described readiness as a moving target shaped by (a) infant clinical stability, (b) caregiver competence with routine and technology-related care, and (c) the availability of practical and emotional supports once the family leaves the protected NICU environment.^9,10,25^ Some studies further reinforce that discharge is more than being medically ready, but a milestone for the infants that depends on physiologic criteria plus caregiver capacity and follow-up infrastructure, particularly for infants with home oxygen, tube feeding, and complex chronic needs.^28,32,36,40^

### 4.1 Interpreting the evidence across themes

#### 4.1.1 Communication and tailoring support to family need

Parents highlighted the importance of clear communication, role clarity, confidence, emotional support, and planning for practical burdens.^9,10,19^ Importantly, early intervention evidence suggests that providing more support is not automatically beneficial, formal transitional support improved adaptation primarily for mothers with higher support needs, while potentially carrying costs (e.g., dependency, strain, or mismatch) when need was lower.^18^ Taken together, these findings argue for stratified, needs-based discharge support, rather than one-size-fits-all education bundles.

#### 4.1.2 Building caregiver judgment: symptom interpretation and home monitoring support

Discharge from NICU often transfers complex surveillance and decision-making from clinicians to parents. This is evident in home oxygen support for BPD transitions,^30,32,33,36^ as well as feeding-related care and post-surgical monitoring.^12,13,15,35^ Parents described persistent vigilance and uncertainty at home, with increased anxiety when instructions were inconsistent, when alarms/monitoring were ambiguous, or when community clinicians lacked neonatal-specific confidence.^11,23,24^ A feasible mitigation strategy is a post-discharge safety-net pathway—early telehealth check-ins, a single neonatal-informed point of contact, and standardized escalation guidance to reduce uncertainty, improve cross-setting alignment, and share surveillance decisions rather than transferring them entirely to families. Although NICU-to-home transition programs exist in some hospitals^44,45^, they remain variable and inconsistently resourced. Many are not designed to support medically complex infants and are more commonly targeted to lower-complexity needs (e.g., feeding tubes or medication weaning), limiting scalability and equity of support.

Practice-focused evidence highlights that caregiving competence is not merely procedural. Parents of infants discharged on oxygen reported that what they needed most was help interpreting symptoms, adjusting to the “new normal,” and knowing when something was serious enough to seek professional care—needs that also show up across studies of chronic lung diseases and other complex discharges.^10,17,22^ The CHD education literature similarly emphasizes anticipatory guidance and recognition of deterioration rather than only task completion,^13^ and structured home monitoring tools aim to operationalize that vigilance in safer, clearer ways.^39,46,47^ However, comparable structured monitoring pathways are far less established for many other groups of medically complex infants, leaving families to rely on informal judgment and variable outpatient support.

#### 4.1.3 System variability across settings and specialties

Multiple papers converge on the idea that discharge breakdowns often occur at handoffs: between NICU teams and outpatient clinicians, between specialties, and between hospital-based recommendations and real-world family capacity^20,28,38^ Care coordination models and structured communication approaches attempt to reduce this issue. For example, a pilot NICU–primary care video-conference model illustrates how intentional, shared planning can address gaps in follow-up understanding and role alignment at the point of transition.^27^ Broader program evaluations similarly suggest families value relational continuity, proactive problem-solving, and a single navigable point of contact after discharge.^20^

However, system variability still persists. Primary care perspectives highlight wide differences in oxygen/feeding management approaches and clinician comfort, particularly for the most medically complex infants, suggesting that discharge planning must actively anticipate outpatient variability rather than assuming downstream standardization.^26^ Attendance barriers for high-risk specialty follow-up (e.g., severe BPD) further show that even well-designed discharge recommendations can fail if transportation, scheduling, caregiver burden, or health system navigation barriers are not addressed.^38^

#### 4.1.4 Discharge as a work-system: system engineering perspectives

Families described discharge as a transition into isolation and resource limitations, where social support, financial stability, and community services determined whether discharge plans were sustainable.^17,24,29,31^ This emphasizes that even a clinically complete discharge plan can be difficult to implement when it does not consider the work system factors in the patient journey rather implicitly assumes stable housing, flexible employment, reliable transportation, sufficient caregiving support, and the capacity to understand and act on complex instructions. Applying SEIPS 3.0,^48^ NICU discharge planning is better framed as a work-system problem in which outcomes emerge from the interaction of people, tasks, tools/technologies, organizational processes, and the external home or community environment across the medically complex infant care journey. Studies of parents caring for medically complex infants also emphasize how fear, hope, couple dynamics, and identity shifts persist well beyond discharge, suggesting the need to broaden planning beyond immediate post-discharge tasks.^19,21,25^ However, despite a broader literature on CMC care needs and caregiver support,^49,50^ far less is known about fragile neonatal populations, where families are often navigating high-complexity home care for the first time, with limited experience and uneven support systems.

### 4.2 Emerging trials fitting into the themes

The three registered clinical trials align strongly with the family empowerment and care continuity strand of this literature:

- Empowerment education with written/manual and multimedia materials maps to interventions targeting caregiver self-efficacy and discharge preparedness—consistent with the readiness domains parents describe as central.^9,41^
- Multi-session discharge-planning plus weekly post-discharge calls maps to structured transitional care/coordination models, echoing the value families place on follow-up reassurance and navigable support after leaving the NICU.^11,20,42^
- Web-based video communication prior to neonatal back-transport maps to communication quality and psychological preparation at transition points—similar to the coordination gaps targeted in NICU–primary care conferencing work.^27,43^

Because these trials are registry-reported here, they also highlight a broader gap in the evidence base: comparatively few rigorous, published randomized evaluations directly test discharge planning models using outcomes that matter to families (confidence, burden, mental health), alongside clinical endpoints (readmissions, ED use, follow-up completion).

### 4.3 Implications for discharge planning practice

NICU discharge planning should be organized as a tailored transition process, not a one-time event or a standard education packet. The evidence supports stratifying discharge support by family need, some families benefit from more intensive coaching and follow-up, while others may experience added strain or mismatch if support is excessive or poorly targeted. In practice, this means pairing core discharge education with a brief needs assessment of medical complexity, caregiver capacity, and home environment to determine the level and type of support. Because discharge shifts surveillance and judgment to parents, planning should move beyond training toward building caregiver decision-making: interpreting symptoms, understanding what is expected vs concerning, and having clear understanding of when to seek help. A pragmatic approach is a post-discharge safety-net pathway—early telehealth/phone check-ins, one neonatal-informed point of contact, and standardized escalation guidance—so monitoring decisions are shared and consistent across settings. Finally, discharge processes should explicitly address handoffs and outpatient variability through clear communication with primary care or specialists and realistic follow-up plans that account for barriers such as transportation, scheduling, caregiver workload, and health system navigation.

## 5. Implications for research

Future work should evaluate needs-based discharge models, including which components are effective for which families and whether there are unintended harms when support intensity does not align with need. Studies should also develop outcomes and measures that reflect caregiver judgment and uncertainty management (symptom interpretation, escalation behavior, confidence), not only procedural competency. There is a clear research gap in structured home monitoring pathways outside CHD—work is needed to adapt monitoring, thresholds, coaching, and follow-up systems for other medically complex neonatal groups (e.g., BPD with oxygen, feeding/growth complexity, post-surgical infants) and to test effects on safety events, emergency department visits, readmissions, caregiver burden, and equity. Using SEIPS 3.0, NICU discharge planning for medically complex infants should be framed as a work-system problem—one that considers how people, tasks, tools/technologies, workflows, and coordination processes interact with the home and community environment over time, especially for fragile neonatal populations where parents are delivering high-complexity care for the first time.

## 6. Conclusion

NICU discharge is best understood as a longitudinal transition, not a single event. Across studies, readiness depended on more than clinical stability or task completion, it also required caregiver confidence and judgment, reliable handoffs, and discharge plans that fit real-world family capacity. Breakdowns were most common at transitions to outpatient care and when guidance, roles, or follow-up pathways were unclear. These findings support shifting from standardized education bundles to needs-based discharge support, stronger cross-setting coordination, and structured post-discharge safety nets that share surveillance decisions with families. Despite growing literature on children with medical complexity, key evidence gaps remain for fragile neonatal infants whose parents are managing high-complexity care for the first time. Advancing discharge practice will require integrated, system-level approaches and research that centers caregiver judgment, longitudinal support, and sustainable access to follow-up care.

## Supplementary Files

This is a list of supplementary files associated with this preprint. Click to download.

- Appendix.docx
